# Mutational profile of rare variants in inflammasome-related genes in Behçet disease: A Next Generation Sequencing approach

**DOI:** 10.1038/s41598-017-09164-7

**Published:** 2017-08-16

**Authors:** Sergio Burillo-Sanz, Marco-Antonio Montes-Cano, José-Raúl García-Lozano, Lourdes Ortiz-Fernández, Norberto Ortego-Centeno, Francisco-José García-Hernández, Gerard Espinosa, Genaro Graña-Gil, Juan Sánchez-Bursón, María Rosa Juliá, Roser Solans, Ricardo Blanco, Ana-Celia Barnosi-Marín, Ricardo Gómez De la Torre, Patricia Fanlo, Mónica Rodríguez-Carballeira, Luis Rodríguez-Rodríguez, Teresa Camps, Santos Castañeda, Juan-Jose Alegre-Sancho, Javier Martín, María Francisca González-Escribano

**Affiliations:** 1 Department of Immunology, Hospital Universitario Virgen del Rocío (IBiS, CSIC, US), Sevilla, 41013 Spain; 2 Department of Internal Medicine, Hospital Clínico San Cecilio, Granada, 18003 Spain; 3 Department of Internal Medicine, Hospital Universitario Virgen del Rocío, Sevilla, 41003 Spain; 4 Department Autoimmune Diseases, Hospital Universitari Clínic, Barcelona, 08036 Spain; 5 Department of Rheumatology, Complejo Hospitalario Universitario A Coruña, A Coruña, 15006 Spain; 6 Department of Rheumatology, Hospital Universitario de Valme, Sevilla, 41014 Spain; 7 Department of Immunology, Hospital Universitari Son Espases, Palma de Mallorca, 07120 Spain; 8 Department of Internal Medicine, Autoimmune Systemic Diseases Unit, Hospital Vall d’Hebron, Universidad Autonoma de Barcelona, Barcelona, 08035 Spain; 9 Department of Rheumatology, Hospital Universitario Marqués de Valdecilla, Santander, 39008 Spain; 10 Department of Internal Medicine, Complejo Hospitalario Torrecárdenas, Almería, 04009 Spain; 11 Department of Internal Medicine, Hospital Universitario Central de Asturias, Asturias, 33011 Spain; 12 Department of Internal Medicine, Hospital Virgen del Camino, Pamplona, 31008 Spain; 13 Deparment of Internal Medicine, Hospital Universitari Mútua Terrassa, Terrassa, 08221 Spain; 14 Department of Rheumatology, Hospital Clínico San Carlos, Madrid, 28040 Spain; 15 Department of Internal Medicine, Hospital Regional Universitario de Málaga, Málaga, 29010 Spain; 16 Department of Rheumatology, Hospital de la Princesa, IIS-Princesa, Madrid, 28006 Spain; 17 Department of Rheumatology, Hospital Universitario Doctor Peset, Valencia, 46017 Spain; 18 Instituto de Parasitología y Biomedicina “López-Neyra”, CSIC, PTS Granada, Granada, 18016 Spain

## Abstract

Behçet’s disease (BD) is an immune-mediated systemic disorder with a well-established association with HLA class I and other genes. BD has clinical overlap with many autoinflammatory diseases (AIDs). The aim of this study was to investigate the role of rare variants in seven genes involved in AIDs: *CECR1*, *MEFV*, *MVK*, *NLRP3*, *NOD2*, *PSTPIP1* and *TNFRSF1A* using a next generation sequencing (NGS) approach in 355 BD patients. To check global association of each gene, 4 tests: SKAT, CollapseBt, C(α) and weighted KBAC were used. Databases: 1000 Genomes Project Phase 3, Infevers, HGMD and ClinVar and algorithms: PolyPhen2 and SIFT were consulted to collect information of the 62 variants found. All the genes resulted associated using SKAT but only 3 (*MVK*, *NOD2* and *PSTPIP1*) with C(α) and weighted KBAC. When all the genes are considered, 40 variants were associated to AIDs in clinical databases and 25 were predicted as pathogenic at least by one of the algorithms. Including only *MVK*, *NOD2* and *PSTPIP1*, the associated to AIDs variants found in BD were 20 and the predicted as pathogenic, 12. The maxima contribution corresponds to *NOD2*. This study supports influence of rare variants in genes involved in AIDs in the pathogenesis of BD.

## Introduction

Behçet’s disease (BD) [OMIM #109650] is a complex and immune-mediated systemic syndrome first described in 1937 by a Turkish dermatologist, Hulusi Behçet^1^. The disease is characterised by inflammatory lesions of blood vessels which lead to a wide range of clinical phenotypes such as recurrent oral and genital ulceration, ocular involvement (mainly uveitis) and skin lesions, amongst others. The aetiology of BD remains obscure, although some evidences suggest that certain infectious agents and environmental factors may trigger the disease in genetically predisposed individuals^2^. BD is a rare disease, more common along the old “Silk Road”; from China to the Mediterranean area^3^. The geographical distribution, along with the familial aggregation and association with HLA class I molecules, currently support the genetic component of BD. In this sense, the HLA genetic contribution is estimated to be 20%^4^, with HLA-B51 having an OR of 5.8^5^. Recently, new non-HLA susceptibility genes, such as *IL23R* and *IL10*, have been reported to be involved in the susceptibility to the disease. Other evidences support the involvement of innate immunity and inflammation in the pathogenesis of the disease, in this sense, overexpression of pro-inflammatory cytokines, such as IL-1, IL-17, IL-23 and IFN-γ has been found in BD patients with active uveitis^6^.

Genetic studies have provided evidence of the key role that inflammasome-related genes play in the pathogenesis of autoinflammatory diseases (AIDs). Inflammasome is a multimeric protein complex which is assembled by members of the families Nod-like receptors (NLR) and AIM2-like receptors (ALR), such as NLRP3, which is the most extensively studied inflammasome, to initiate defense mechanisms against bacteria. Two signals are required for NLRP3 activation: induction of the *NLRP3* gene expression by sensor proteins (like TLRs and NOD2) and activation of the protein by a pathogen-associated molecular pattern (PAMP) or damage-associated molecular pattern (DAMP). NLRP3 activation triggers NLRP3-inflammasome assembly which results in IL1β release and pyroptosis^7^. The AIDs affect different organs and systems being some of them monogenic disorders and, therefore, variants in inflammasome-related genes cause the disease. Thus, variants in the *MEFV* gene cause familial mediterranean fever (FMF, OMIM #249100); in *NLRP3*, cryopyrin-associated periodic syndrome (CAPS, OMIM #120100); in *TNFRSF1A*, TNF receptor-associated periodic syndrome (TRAPS, OMIM#142680); in *MVK*, Hyper-IgD syndrome (HIDS, OMIM#249100) and in *PSTPIP1*, pyogenic arthritis, pyoderma gangrenosum, and acne syndrome (PAPAS, OMIM#604416). Nevertheless, there are also some syndromes classified as AIDs, which are polygenic and complex, such as Crohn’s disease (CD, OMIM #266600), in these cases, the disease is not caused by variants in a specific gene, but it is associated to changes in several genes, for example, CD has been associated to variants of the *NOD2* among other genes. In addition, environmental factors are required in genetically susceptible individuals to trigger these complex diseases.

BD has a considerable clinical overlap with many of the AIDs, therefore it is plausible to hypothesize that variants in genes involved in these diseases, most of them related with the inflammasome, could partly explain the genetic component of BD. The involvement of these genes in BD could have remained hidden in genome wide association studies (GWAS) because, although these studies are effective in the identification of common variants associated to diseases, they are not suitable for detecting associations with rare or infrequent variants. In this sense, the study of rare variants and the study of polymorphisms have different approaches because the case-control studies, usually used to compare distributions in order to investigate the involvement of polymorphisms in susceptibility to complex diseases, are unusefull to analyze rare variants, because of the absence or the extremely low frequency of their minor allele in general population.

The aim of this study was to assess whether rare variants in genes involved in AIDs, most of them related with the inflammasome, contributes to the pathogeny of BD. The study was performed by exploring the mutational spectrum of this set of genes in a cohort of BD patients using a next generation sequencing (NGS) approach.

## Methods

### Patients

The study population consisted of 355 patients diagnosed with BD (43.7% males) of Spanish European descent. Peripheral blood (PB) samples collected in EDTA tubes were obtained of patients from different Spanish hospitals^8^, all of them fulfilled the 1990 International Study Group classification criteria for this disease^9^. Clinical features of the patient group were: 100% had oral ulcers, 75.3% skin lesions, 59.5% genital ulcers, 54% uveitis, 42% arthritis, 21% vascular, 18.2% neurological, 16.4% positive pathergy test and 15.5% gastrointestinal involvement and 36% were B51 positive. The study was approved by the ethical committees of all centres and hospitals involved (Hospital Universitario Virgen del Rocío, Hospital Clínico San Cecilio, Hospital Universitari Clínic, Complejo Hospitalario Universitario A Coruña, Hospital Universitario de Valme, Hospital Universitari Son Espases, Hospital Vall d’Hebron, Hospital Universitario Marqués de Valdecilla, Complejo Hospitalario Torrecárdenas, Hospital Universitario Central de Asturias, Hospital Virgen del Camino, Hospital Universitari Mútua Terrassa, Hospital Clínico San Carlos, Hospital Regional Universitario de Málaga, Hospital de la Princesa, Hospital Universitario Doctor Peset, Instituto de Parasitología y Biomedicina López-Neyra). All participants signed a written informed consent prior to their enrolment in the study.

### Sample preparation

Genomic DNA was extracted from 200 to 400 µl of PB using ‘QIamp DNA mini kit’ (Qiagen, Barcelona, Spain) following manufacture’s recommendations. DNA preparations were quantified in a Qubit® 3.0 fluorometer and diluted to 0.67 ng/µl. For convenience, DNA samples obtained from 355 patients were pooled at equimolar concentration in 71 pools (5 samples/pool). Each pool was processed as a single sample in the subsequent sequencing procedures.

### Next Generation Sequencing panel-based analyses

A custom-designed NGS gene panel (AmpliSeq™ software, Ion Torrent, Thermo Fisher Scientific, Waltham, MA) targeting all the coding regions and flanking intronic sequences of 7 AIDs-related genes was used in this study: *NLRP3* (NG_007509.2)*, TNFRSF1A* (NG_007506.1)*, MVK* (NG_007702.1)*, PSTPIP1* (NG_007526.1)*, MEFV* (NG_007871.1)*, NOD*2 (NG_007508.1) and *CECR1* (NG_033943.1). The total target size was 27,375 bases, distributed in 125 amplicons with a length of 219 ± 41 bases. After preparation of pools, the amplification and barcoding were carried out with the Ion AmpliSeq™ Kit for Chef DL8, according to the manufacturer´s recommendations, and it consisted on 19 cycles of PCR in an Ion Chef system. Libraries were quantified by qPCR or with Qubit® 3.0 Fluorometer, diluted to a final concentration of 40 pM and used for template preparation with the Ion PGM™ Hi-Q™ Chef Kit on the Ion Chef system with 200 bases of templated size. Sequencing templates were loaded in Ion 318™v2BC Chips (8 pools labeled with barcodes were loaded in each chip) and sequenced in an Ion PGM™ Sequencer. The total readings/amplicon were 4,066 ± 1,483 bases and 99.3% amplicons had a coverage greater than 100X.

### Variant calling and allele quantification

Variant calling was performed with Ion Reporter™ Software v5.0^10^, using a somatic mutation detection and quantification pipeline implemented in the software. Readings were aligned to the human genome reference, hg19. Variants reported by Ion Reporter were verified and filtered by visual inspection on Integrative Genomics Viewer IGV v2.3.68, Broad Institute^11^ in order to verify the missense mutation presence and to perform a confirmation of the reading alignments. Ion Reporter was also used to quantify the number of mutated alleles in each pool. In our study, one variant in a pool corresponds to 1 allele out of the 10 possible (5 patients per pool) and therefore, it would be quantified as 10%.

### Validation of the sample pooling results

Ion Reporter was used to quantify the number of mutated alleles/pool (namely, to quantify germline alleles in pooled samples). This software was primary designed to quantify somatic variants in individual samples; therefore, a validation study was carried out for assessing the accuracy of the method. This validation was performed by analyzing 101 variants in samples of 5 patients which had previously been sequenced individually. The quality control results were 100% sensitivity, all variants detected in the analysis of the individual samples were also detected in pooled samples and 100% specificity, none false-positives were present in the analysis of the pools. The global correlation in the allele quantification was R^2^ = 0.91 (Figure S2).

### Classification of variants

Variants found in patients were classified according to their minor allele frequency (MAF) in the entire population of the 1000 Genomes Project Phase 3 (www.ensembl.org) as common, those variants with a MAF > 0.05; rare, 0 < MAF < 0.05; and undetected, undescribed in that database. Clinical significance of the missense variants found was investigated in human mutation databases: Infevers^12^, HGMD^13^ and ClinVar^14^ in order to know, which variants are considered non-pathogenic polymorphisms, which variants have been described to be associated with AIDs and which variants are not described. Lastly, two bioinformatics approximations, PolyPhen2 (HumVar model) and SIFT, were used to evaluate the functional impact of variants in the proteins^15^.

### Disease association studies

The association of the rare variants of each gene with the diseases was evaluated for using Variant Tools software^16, 17^. For patient data, the 71 calling files from Ion Reporter were randomly splitted in 5 files with GATK (Broad Institute, MA) to conserve number of samples (n = 355), these VCF files were imported to the Variant Tools software. For the controls, data from 107 samples of 1000 genomes project IBS population were retrieved as VCF files using Data Slicer^18^, according to bed file containing target regions of our NGS panel. These files were concatenated with the VCF tools software^19^ and imported as a single file to Variant Tools. Variant annotation was filtered with NSFP database, including only missense variants with MAF ≤ 0.05 in controls. These variants were included in the gene association studies performed and analyzed with the Variant Tools implementation of 4 different tests: a burden or aggregation test, SNP-set (Sequence) Kernel Association Test (SKAT)^20^; a fixed threshold collapsing method, CollapseBt^21^; one test used to contrast the unusual distribution of minor alleles in cases and controls, C(α)^22^; and, lastly, a weighted burden test^23^, weighted KBAC^24^, which is similar to SKAT but scoring the variants taking into account their Polyphen2 score. Variants were grouped by gene name in refGene database. NOD2 p.Arg702Trp and p.Gly908Arg single variant association to BD was evaluated by Chi-square test. A p-value < 0.05 was considered significant.

Association between rare variants (those with MAF < 0.05) of each gene and clinical manifestations was investigated after Sanger-sequencing of all the samples of the pool having each rare specific variant in order to know which sample has the variant. Ditribution were compared using Chi-square test with Bonferroni’s correction, a p_c_-value < 0.05 was considered significant.

### Sanger sequencing verification of novel variants

Presence of those variants that were not found in human mutation databases and rare variants (those with MAF < 0.05) was verified by Sanger sequencing with BigDye™ Direct Cycle Sequencing kit using primers generated by Thermo Fisher NGS Sanger confirmation software on Ion Reporter, following manufacturer’s recommendations. Patients carrying each variant were identified in all the pools using this method. The novel Sanger-verified variants (*CECR1* p.Arg49Trp, p.Ala247Val; p.Met309Ile and p.Val349Ile, *MEFV* p.Gly111Glu, p.Leu367Val; and p.His404Arg, *PSTPIP1* p.Thr68Met, p.Val122Ile p.Asp289His and; *NOD2* p.Leu81Val, p.Leu349Phe, p.Val733Leu and p.Val816Ile, and *TNFRSF1A* p.His155Tyr, p.Ile199Thr, p.Arg312Lys and p.Asn336His) were submitted to Infevers and ClinVar databases.

### Data Availability

Complete datasets generated during and/or analyzed during the current study are available from the corresponding author on reasonable request. All the NGS raw sequence reads have been deposited in SRA database at: https://www.ncbi.nlm.nih.gov/Traces/study/?acc = SRP105162 New variants have been deposited in ClinVar database at: https://www.ncbi.nlm.nih.gov/clinvar/submitters/505967/

### Ethical use of human participants statement

All participants signed a written informed consent to publish identified information prior to their enrolment in the study. All methods were performed after obtaining the signed informed consent from patients, and in accordance to our institution (Hospital Universitario Virgen del Rocio) ethical committee, and with the Helsinki Declaration of 1975, as revised in 1983.

## Results

**Figure 1:**
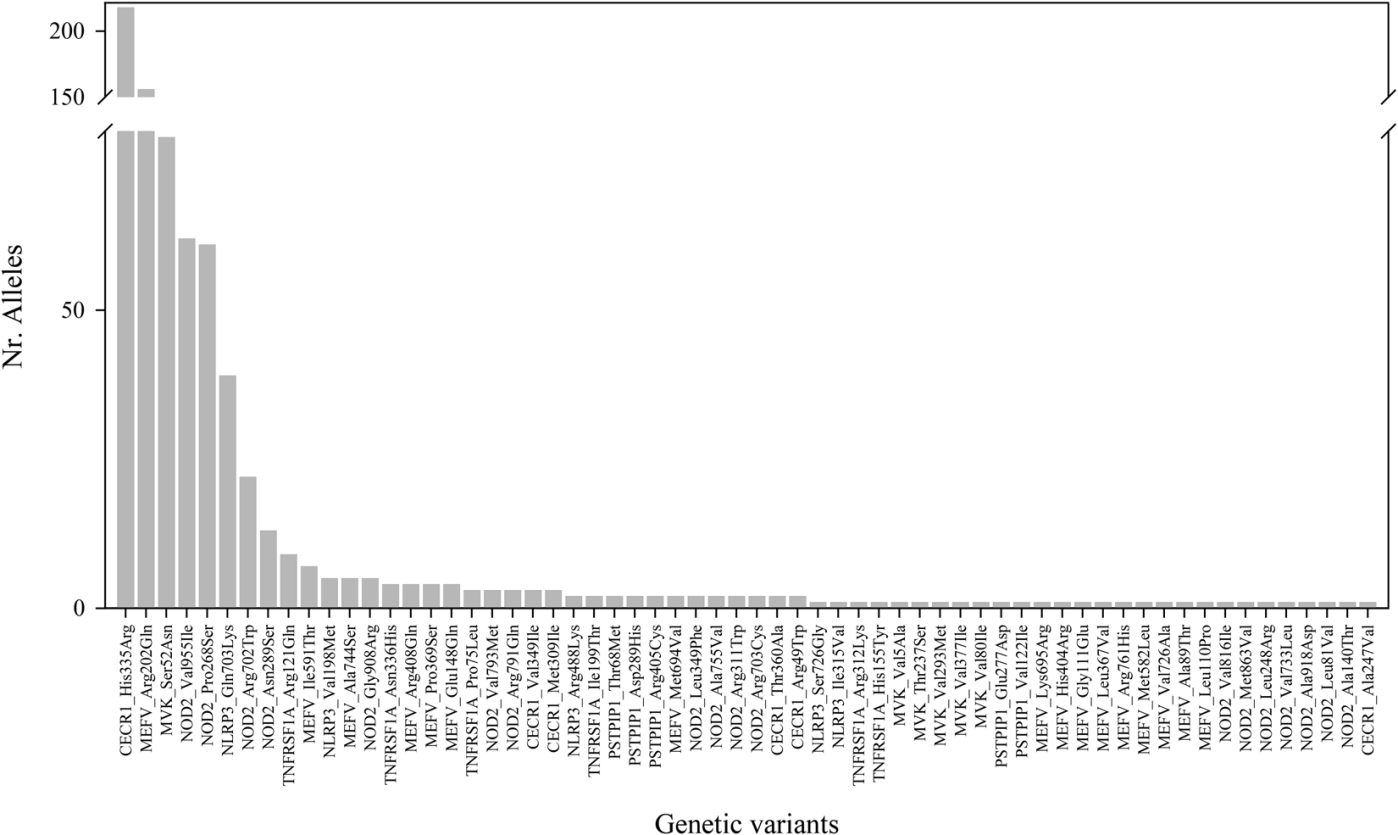
Graphical representation of the number of alleles (y-axis) of each variant detected (listed in x-axis and ordered according to the number of alleles found) in our Behçet disease patient cohort.

All the coding regions and flanking intronic sequences of the 7 genes studied were covered with two exceptions: the amplicon of the first half of the *MEFV* exon 5, (aminoacids from 453 to 486) because it did not reach the minimal coverage requirements and a region in *TNFRSF1A* exon 10 (aminoacids 372 and 380) which was not targeted by the primers proposed by the Ion AmpliSeq™ software. In the total of 27,375 bases sequenced in these 7 genes, 62 missense (exonic) single nucleotide variants (SNP) were found (detailed information is displayed in Table S1). Variants were detected in all the genes and the frequency of each of them is displayed in Fig. 1. Neither insertions/deletions with enough quality of sequence, nor intronic variants in the two exon-nearest bases were identified.

Five variants were common SNP (MAF > 0.05): 1 in *CECR1*, 2 in *MEFV*, 1 in *MVK* and 1 in *NOD2*; but most of them are rare: 34 had MAF between 0 and 0.05: 2 in *CECR1*, 7 in *MEFV*, 3 in *MVK*, 4 in *NLRP3*, 14 in *NOD2*, 1 in *PSTPIP1* and 3 in *TNFRSF1A;* lastly, 23 variants (including 7 new) had a frequency equal to 0 in the entire population of the 1000 Genomes database: 3 in *CECR1*, 7 in *MEFV*, 2 in *MVK*, 1 in *NLRP3*, 4 in *NOD2*, 3 in *PSTPIP1* and 3 in *TNFRSF1A* (Table 1 and Figure S2A). Rare and not reported variants (MAF < 0.05) were considered to analyze the association at gene level taking the IBS population of the 1000 genomes project as control. The results with the 4 tests used are displayed in Table 2 and they are different depending on the test applied: the seven genes could be evaluated and resulted associated with SKAT; when CollapseBt was used, *CECR1* had to be excluded from the analysis and other two genes resulted non-associated: *MEFV* and *TNFRSF1A*; lastly, the results with the C(α) and weighted KBAC tests were the same, *CECR1* could not be evaluated and *MEFV*, *NLRP3* and *TNFRSF1A* did not reach significance whereas *MVK*, *PSTPIP1* and *NOD2* were associated.

**Table 1:** Classification of the variants found in the present study in BD patients. The number of variants in each group is displayed.

Gene	**Frequencies of variants in 1000 Genomes All Populations Database**																	
	MAF > 0.05						0 < MAF < 0.05						MAF = 0					
	**Human Mutation Databases**						**Human Mutation Databases**						**Human Mutation Databases**					
	Non-Pathogenic		Associated to other syndromes		Unknown		Non-Pathogenic		Associated to other syndromes		Unknown		Non-Pathogenic		Associated to other syndromes		Unknown	
CECR1	1		0		0		0		0		2		0		1		2	
MEFV	1		1		0		0		7		0		0		4		3	
MVK	1		0		0		0		3		0		0		2		0	
NLRP3	0		0		0		1		3		0		0		1		0	
NOD2	1		0		0		1		12		0		0		1		4	
PSTPIP1	0		0		0		0		1		1		0		1		2	
TNFRSF1A	0		0		0		0		2		1		0		0		3	
Total	4		1		0		2		28		4		0		9		14	
	**PolyPhen2 prediction**																	
	**Damaging**						**Damaging**						**Damaging**					
	Yes	No	Yes	No	Yes	No	Yes	No	Yes	No	Yes	No	Yes	No	Yes	No	Yes	No
	0	4	1	0	0	0	0	2	12	16	1	3	0	0	3	6	3	11
	**SIFT prediction**																	
	**Damaging**						**Damaging**						**Damaging**					
	Yes	No	Yes	No	Yes	No	Yes	No	Yes	No	Yes	No	Yes	No	Yes	No	Yes	No
	0	4	1	0	0	0	0	2	7	21	0	4	0	0	2	7	5	9

**Table 2:** Analysis of the association with BD of rare variants in seven genes related to AIDs.

Gene	P-values			
	^1^SKAT	^1^CollapseBt	^2^C(α)	^2,3^Wighted KBAC
*CECR1*	**3.08E-06**	NA	NA	NA
*MEFV*	**0.028**	0.35	0.786	0.47
*MVK*	**9.49E-05**	**3.49E-12**	**0.0004**	**0.00039**
*NLRP3*	**0.020**	**0.036**	0.078	0.064
*NOD2*	**0.002**	**0.0008**	**0.038**	**0.0014**
*PSTPIP1*	**0.0001**	**0.01**	**0.008**	**0.016**
*TNFRSF1A*	**0.0001**	0.821	0.73	0.65

These tests analyze the association of rare variants (MAF < 0.05) grouped by gene.

^1^Asymptotic and^2^ permuted P-values.^3^Variants weighted by their Polyphen2 score.

The P-values < 0.05 were considered significant and they are shown in bold.

NA: Not available, the gene could not be evaluated.

In regard to the available clinical information in human mutation databases, 4 out of the 5 missense variants with MAF > 0.05 in healthy population are considered non-pathogenic polymorphisms (*CERC1* p.His335Arg, *MEFV* p.Arg202Gln, *MVK* p.Ser52Asn and *NOD2* p.Pro268Ser), whereas one is considered associated to FMF (*MEFV* p.Glu148Gln). Regarding the 34 variants with MAF between 0 and 0.05 in healthy population, 2 have been described as non-pathogenic polymorphisms although functional effects have been reported in some cases^25–27^ (*NLRP3* p.Glu703Lys and *NOD2* p.Val955Ile, MAF in healthy population 0.02 and 0.03, respectively), 28 have been associated with different AIDs and 4 are not found in any clinical database or report. Finally, regarding those 23 variants do not detected in healthy population, 9 have been associated with AIDs, and 14 have not been associated with any disease neither in clinical databases nor in journal publications, 7 of which are new (not found in any sequencing database): *MEFV* p.Leu367Val, *NOD2* p.Val733Leu and p.Val816Ile, *PSTPIP1* p.Val122Ile and p.Asp289His and *TNFRSF1A* p.His155Tyr and p.Asn336His (Table 1 and Figure S2B).

Next, two bioinformatic approaches, PolyPhen2 and SIFT, were used to evaluate the functional impact of variants in the proteins. PolyPhen2 predicted some damaging variant in all the 7 genes included, whereas according to SIFT, none of the variants detected in our BD patients in *MVK*, *NLRP3* and *PSTPIP1* were damaging. A total of 25 out of the 62 variants (38.7%) detected were predicted as damaging, 10 only by PolyPhen2, 5 only by SIFT and 10 by both approaches. Both algorithms, predicted *MEFV* p.Glu148Gln as damaging, but the rest of the 4 SNPs with MAF > 0.05 in healthy populations and also *NLRP3* p.Glu703Lys and *NOD2* p.Val955Ile as non-damaging. Regarding rare variants associated to AIDs in clinical databases or reports, the prediction of PolyPhen2 and SIFT was damaging for 41% and 24% respectively, whereas for the unknown variants, it was 22% and 27% (Table 1 and Table S1). With respect to the damaging prediction for the 10 variants described in healthy population but not found in clinical databases or reports, 2 were predicted by PolyPhen2: *CECR1* p.Ala247Val and *PSTPIP1* p.Thr68Met, 2 by SIFT: *CECR1* p.Arg49Trp and *NOD2* p.Leu81Val and 1 for both: *MEFV* p.His404Arg. In regards to the prediction for the 7 new variants, 1 was predicted as damaging by PolyPhen2, *PSTPIP1* p.Asp289His, whereas SIFT predicted 2: *NOD2* p.Leu349Phe and *TNFRSF1A* p.Asn336His.

Lastly, to evaluate the contribution to the disease of these 7 genes, the percentage of pathogenic alleles in our BD patients was calculated taking into account the results of: 1) the gene association tests, 2) the variants included in clinical databases associated to AIDs, and 3) the prediction of PolyPhen2 and SIFT. When all the 40 variants associated to AIDs in clinical databases in these 7 genes are included in the estimation, the total number of mutated alleles in our patients is 219 (out of 710 possible, 30.84%). The maxima contribution corresponds to *NOD2* (16.61% of the variants), followed by *NLRP3* (6.76%), *MEFV* (4.4%) and the rest, *TNFRSF1A* (1.69%), *MVK* (0.70%) and *PSTPIP1* (0.42%) and *CECR1* (0.28%). When only the *MVK*, *NOD2* and *PSTPIP1* are included, the number of mutated alleles in the 20 variants reported as associated to AIDs is 126 (17.75%). A total 71 alleles (10.0%) were predicted as pathogenic by PolyPhen2 and/or SIFT but only 44 alleles (6.20%) were predicted by the two algorisms, corresponding the maxima contribution to *NOD2* in all the cases (Table 3).

**Table 3:** Number of alleles of the different variants found in our BD patients reported in clinical database and/or pathogenic according to PolyPhen2 and/or SIFT.

**Variant**	**Number of alleles in BD patients (Total of alleles 710)**	**AID**
**CECR1_Arg49Trp** ^**1**^	2	Unknown
**CECR1_Ala247Val** ^**2**^	1	Unknown
**CECR1_Thr360Ala** ^**3**^	2	Sneddon’s syndrome
**MEFV_Ala89Thr** ^**3**^	1	FMF
**MEFV_Leu110Pro** ^**1**^	1	FMF
**MEFV_Glu148Gln** ^**3**^	5	FMF
**MEFV_Pro369Ser** ^**3**^	3	FMF
**MEFV_His404Arg** ^**3**^	1	Unknown
MEFV_Arg408Gln	3	FMF
MEFV_Met582Leu	1	Recurrent Arthritis, FMF
MEFV_Ile591Thr	7	FMF
MEFV_Met694Val	2	FMF
**MEFV_Lys695Arg** ^**2**^	1	FMF
MEFV_Val726Ala	1	FMF
MEFV_Ala744Ser	5	FMF
MEFV_Arg761His	1	FMF
MVK_Val5Ala	1	HIDS
MVK_Val80Ile	1	HIDS
**MVK_Thr237Ser** ^**2**^	1	HIDS
**MVK_Val293Met** ^**2**^	1	HIDS
MVK_Val377Ile	1	HIDS
NLRP3_Val198Met	5	FCAS
NLRP3_Ile315Val	1	MAGIC Syndrome
NLRP3_Arg488Lys	2	FCAS
NLRP3_Gln703Lys	39	FCAS
**NLRP3_Ser726Gly** ^**2**^	1	CINCA/NOMID
**NOD2_Leu81Val** ^**1**^	1	Unkown
NOD2_Ala140Thr	1	CD
**NOD2_Leu248Arg** ^**3**^	1	CD
NOD2_Asn289Ser^*^	13	CD
**NOD2_Arg311Trp** ^**3**^	2	CD
**NOD2_Leu349Phe** ^**1**^	2	Unkown
**NOD2_Arg702Trp** ^3*^	22	CD
**NOD2_Arg703Cys** ^**3**^	2	CD
**NOD2_Ala755Val** ^**2**^	2	CD
NOD2_Arg791Gln	3	Spondylarthropathy
NOD2_Val793Met	3	CD
NOD2_Met863Val	1	CD
**NOD2_Gly908Arg** ^3*^	5	CD
**NOD2_Ala918Asp** ^**2**^	1	CD
NOD2_Val955Ile	62	CD
**PSTPIP1_Thr68Met** ^**2**^	2	Unknown
PSTPIP1_Glu277Asp	1	PAPASH
**PSTPIP1_Asp289His** ^**2**^	2	Unknown
PSTPIP1_Arg405Cys	2	Idiopathic juvenile arthritis
**TNFRSF1A_Pro75Leu** ^**2**^	3	TRAPS
TNFRSF1A_Arg121Gln	9	TRAPS
**TNFRSF1A_Asn336His** ^**1**^	4	Unkown

Variations pathogenic according to the bioinformatic prediction are displayed in bold. ^1^SIFT, ^2^PolyPhen2 and ^3^both.

^*^ *NOD2* loss of function variants reported as associated to risk in CD but as possible protective in BD.

*NOD2* p.Asn289Ser, p.Arg702Trp and p.Gly908Arg are loss of function variants which are damaging according with PolyPhen2 and associated to CD, although, they have been reported as possible protective in BD^28, 29^. These variants have a relatively high frequency, thus, in order to improve the knowledge of the effect of these variants in BD, their frequencies in the patient group were compared with those obtained in ethnically matched healthy controls. No significant differences were observed in any case (Table S2). Even excluding the alleles provided by these 3 loss of function variants, *NOD2* remain as the major contributor (11% of the alleles).

Finally, Table S3 summarizes the clinical features of patients with rare variants (MAF < 0.05) in each gene, no statistically significant differences were found in any case (p_c_ > 0.05).

## Discussion

The most relevant result of this study is the description of a considerable number of rare variants, in inflammasome-related genes in patients with a polygenic, complex and immune-mediated disease, such us BD, using a NGS approach. These exonic variants are extremely rare or even absent woldwide in healthy population and most of them are considered causative in monogenic AIDs.

Several studies have investigated the association of BD with specific missense variants in two of these genes, *MEFV* and *NOD2* ^30^. In addition, the entire coding sequence of *NOD2* has also been investigated^28, 29^, although NGS was used only in one study^29^. The previous NGS study, reported no association of any of the 5 of the Japanese studied genes (*NLRP3*, *TNFRSF1A*, *PSTPIP1*, *MEFV* and *NOD2*) but association of the two genes (*MEFV* and *NOD2*) investigated in Turkish. Study of the association of rare variants in complex diseases has difficulties because of the lack of statistical power. In order to avoid this problem, specific tests grouping variants to check the association of the entire gene have been designed. *NOD2*, resulted associated with all the tests used, nevertheless, association of *MEFV* was detected only using SKAT; differences among tests depend on the statistical power, type I errors likehood, linkage disequilibrium (LD), etc. Regarding the rest of the genes studied, association of *PSTPIP1* and *MVK* was detected with all of the tests, whereas non-association of *NLRP3* and *TNFRSF1A* was observed with C(α) and weighted KBAC, finally, *CERC*, had to be removed from these two last tests.

Another point that hinders the address of these studies is to determine which variants could influence the pathology and which not. In general, variants common in healthy population are polymorphism but there are exceptions such as *MEFV* p.Glu148Gln (MAF in global healthy population = 0.13) very common in Asian populations, although rare in European (MAF = 0 in the IBS population) which is found in 14% of Spanish FMF patients^31, 32^. In regard to the rare variants, there is a great diversity and population differences. For example, the previous NGS study reported 27 variants with MAF < 0.03, but only 4 were shared with those found in the present suggesting that these variants are specific of each population. In addition, the low frequency of these variants entails a lack of information in clinical databases and ignorance of functional implications based only in the prediction algorithms available. PolyPhen2 is an algorithm with a high specificity; in fact, in our study it predicts as benign all the variants known to be polymorphisms, but its sensitivity is low, many of the variants associated to AIDs are classified as benign by PolyPhen2, even the highly pathogenic *MEFV* p.Met694Val the most common variant in FMF Spanish patients^32^. Regarding to SIFT, also has a lack of sensitivity, since variants with demonstrated pathogenicity, such as *MEFV* Met694Val and Val726Ala, and *MVK* Thr237Ser and Val377Ile, are predicted as tolerated. Rate of concordance between both algorithms in our study was 75.8% and, although in general, SIFT had a lower sensitivity than Polyphen2, discordances in both senses were found. Combination of both algorithms might be a good option, especially in those many cases in which other functional information is not available. One of the most important limitations of our study is precisely the lack of functional studies and the difficulty to address them easily because of the multiple variants found. Therefore, some of these variants may contribute to the disease onset, but others could be only SNPs without any effect, so that functional and familiar segregation studies of each variant should be carried out in order to assess their phenotypic effects and penetrance. In fact, we can not discard influence of rare variants with clinical features because, although no statistically significant differences were found in any case, sample size is too low to discard association.

In addition, our study used IBS population as control and, although this population is theoretically ethnically matched with our BD cohort, samples were studied in a different way (individually, not pooled) and using a different platform. Therefore, although previous studies suggest that data obtained from both platforms are comparable^33^, and analysis of the distribution of common SNPs along with verification by Sanger sequencing of variants with MAF < 0.05 suggests that this possibility is unlikely, stratification problems can not be completely ruled out.

All these reasons make difficult the quantification of the weight of each gene in the disease, although, *NOD2* was the most contributor with all the conditions used in our study. *NOD2* encodes a protein involved in bacterial recognition and loss of function variants, such as p.Arg702Trp, p.Gly908Arg and p.Leu1007fsinsC, has been associated with CD^34, 35^. These loss of function *NOD2* variants have been reported as possible protective in BD, nevertheless, no evidence of this role was found in our cohort. Additionally, the distribution of the p.Asn289Ser, which is not associated to CD but which has a marked reduction of NF-kB activation^36^, is not different in BD Spanish patients and controls. From these 3 variants, p.Arg702Trp was not included in the association study of the gene because of its MAF > 0.05. Together with *NOD2, PSTPIP1* and *MVK* are associated with the disease with all the tests used. PSTPIP1 is also part of inflammasome interacting with MEFV, variants in this gene cause PAPAS, an autosomic dominant syndrome and PAPAS-related variants enhance the release of IL1β^37^. Regarding *MVK*, it encodes an enzyme involved in cholesterol synthesis. Loss of function variants in this gene gives rise to the accumulation of mevalonic acid and mevalonolactone that co-stimulate inflammasome. The more than 120 variants described to date in this gene have been associated with HIDS and mevalonate kinase deficiency^38–40^. Association of the rest of the genes has a less support in our study, this is the case of *MEFV* which encodes a pyrin that is a component of NLRP3 inflammasome complex^41^. The result of no-association of *MEFV* when CollapseBt was used could be partiallly explained because some of the variants of this gene are described as part of the same complex allele^12, 31^, being the only showing evidences of LD in our study and, as a consequence, some genetic test could overestimate the association of *MEFV*. In addition, discrepancies among populations could be based in ethnical differences. In this sense, previous NGS study in Turkish patients concluded that the non-synonymous *MEFV* variants are, in general, associated to the disease although the main weight was attributed to p.Met694Val, nevertheless, only 2 alleles of this variant were found in our cohort. Additionally, despite the relatively high number of variants found in this gene in both studies, only 8 (including founder *MEFV* variants) are shared. Variants in this gene cause FMF, those better known as high pathogenic are 4: p.Met680Ile, p.Met694Val, p.Met694Ile and p.Val726Ala^42^ that have been reported in BD patients^30, 43, 44^. These variants were found in our cohort together with other 14 variants, including the well-known non-pathogenic polymorphism p.Arg202Gln, although the *MEFV* most common variant in our study (excepting p.Arg202Gln) was p.Ile591Thr, which is an uncommon variant previously associated to FMF^32^. Therefore, further studies are needed to clarify the role of this gene in BD. Association of *NLRP3* was detected with SKAT and CollapseBt but did not reach significance with C(α) and weighted KBAC, perhaps because of lack of statistical power. This gene encodes a component of the inflammasome and its variants have been associated with diverse autoinflammatory syndromes. *TNFRSF1A*, which encodes a TNF receptor and cause TRAPS, was found associated only with SKAT. Lastly, *CECR1*, which could only be analyzed with SKAT, encodes an adenosine deaminase (ADA2) and has been associated with Cat Eye Syndrome, Sneddon’s syndrome and Polyarteritis nodosa. Variants in this gene cause hypogammaglobulinemia and decreased B-cell and monocyte differentiation.

In summary, many rare variants in AIDs related genes are found in BD patients. Some of these variants may play a role in the pathogenesis of this disease, although others could be polymorphisms without any significance in the susceptibility. Specifically, this study supports influence of variants of *NOD2, PSTPIP1* and *MVK* in the pathogenesis of BD. Among these genes, *NOD2* would be the major contributor. Altogether these results support a contribution of the innate immune system in the pathogenesis of BD.

## Electronic supplementary material

Supplementary Material

## References

[1] Behcet H (1937). Uber rezidivierende aphthose, durch ein virus verursachte geschwure am mund, am auge und an den genitalien. Dermatol. Wochenschr..

[2] Mendes D (2009). Behcet’s disease–a contemporary review. J Autoimmun.

[3] Sakane T, Takeno M, Suzuki N, Inaba G (1999). Behçet’s disease. N. Engl. J. Med..

[4] Yazici H, Fresko I, Yurdakul S (2007). Behçet’s syndrome: disease manifestations, management, and advances in treatment. Nat. Clin. Pract. Rheumatol..

[5] de Menthon M, Lavalley MP, Maldini C, Guillevin L, Mahr A (2009). HLA-B51/B5 and the risk of Behçet’s disease: a systematic review and meta-analysis of case-control genetic association studies. Arthritis Rheum..

[6] Chi W (2008). Upregulated IL-23 and IL-17 in Behçet patients with active uveitis. Invest. Ophthalmol. Vis. Sci..

[7] Man SM, Kanneganti T-D (2015). Regulation of inflammasome activation. Immunol. Rev..

[8] Ortiz-Fernández L (2016). Genetic Analysis with the Immunochip Platform in Behçet Disease. Identification of Residues Associated in the HLA Class I Region and New Susceptibility Loci. PLoS One.

[9] Criteria for diagnosis of Behçet’s disease. International Study Group for Behçet’s Disease (1990). Lancet (London, England).

[10] Scientific, T. F. Ion Reporter^TM^ Software. 2015 Available at: https://ionreporter.thermofisher.com/ir/. (Accessed: 8th January 2016).

[11] Robinson JT (2011). Integrative genomics viewer. Nat. Biotechnol..

[12] Sarrauste de Menthière C (2003). INFEVERS: the Registry for FMF and hereditary inflammatory disorders mutations. Nucleic Acids Res..

[13] Institute of Medical Genetics in Cardiff. The Human Gene Mutation Database. Available at: http://www.hgmd.cf.ac.uk/ac/index.php. (Accessed: 1st August 2016).

[14] Landrum MJ (2016). ClinVar: public archive of interpretations of clinically relevant variants. Nucleic Acids Res..

[15] Adzhubei IA (2010). A method and server for predicting damaging missense mutations. Nat. Methods.

[16] Wang GT (2014). Variant association tools for quality control and analysis of large-scale sequence and genotyping array data. Am. J. Hum. Genet..

[17] San Lucas FA, Wang G, Scheet P, Peng B (2012). Integrated annotation and analysis of genetic variants from next-generation sequencing studies with variant tools. Bioinformatics.

[18] 1000 Genomes Data Slicer. Available at: http://browser.1000genomes.org/Homo_sapiens/UserData/SelectSlice. (Accessed: 1st October 2016).

[19] Danecek P (2011). The variant call format and VCFtools. Bioinformatics.

[20] Wu MC (2011). Rare-variant association testing for sequencing data with the sequence kernel association test. Am. J. Hum. Genet..

[21] Li B (2008). Methods for detecting associations with rare variants for common diseases: application to analysis of sequence data. Am. J. Hum. Genet..

[22] Neale BM (2011). Testing for an Unusual Distribution of Rare Variants. PLoS Genet..

[23] Madsen BE, Browning SR (2009). A Groupwise Association Test for Rare Mutations Using a Weighted Sum Statistic. PLoS Genet..

[24] Liu DJ, Leal SM (2010). A Novel Adaptive Method for the Analysis of Next-Generation Sequencing Data to Detect Complex Trait Associations with Rare Variants Due to Gene Main Effects and Interactions. PLoS Genet..

[25] Hedegaard CJ, Enevold C, Sellebjerg F, Bendtzen K, Nielsen CH (2011). Variation in NOD2 augments Th2- and Th17 responses to myelin basic protein in multiple sclerosis. PLoS One.

[26] Blomgran R (2012). Common Genetic Variations in the NALP3 Inflammasome Are Associated with Delayed Apoptosis of Human Neutrophils. PLoS One.

[27] Verma D (2012). The Q705K polymorphism in NLRP3 is a gain-of-function alteration leading to excessive interleukin-1β and IL-18 production. PLoS One.

[28] Ahmad, T. *et al*. CARD15 polymorphisms in Behçet’s disease. *Scand. J. Rheumatol*. **34**, 233–7 (2005). 10.1080/03009740510018714 16134731

[29] Kirino Y (2013). Targeted resequencing implicates the familial Mediterranean fever gene MEFV and the toll-like receptor 4 gene TLR4 in Behcet disease. Proc. Natl. Acad. Sci..

[30] Baruch Y (2011). MEFV, TNFRSF1A and CARD15 mutation analysis in Behcet’s disease. Clin Exp Rheumatol.

[31] Bernot A (1998). Non-founder mutations in the MEFV gene establish this gene as the cause of familial Mediterranean fever (FMF). Hum. Mol. Genet..

[32] Touitou I (2001). The spectrum of Familial Mediterranean Fever (FMF) mutations. Eur. J. Hum. Genet..

[33] Erguner B, Ustek D, Sagiroglu MS (2015). Performance comparison of Next Generation sequencing platforms. Conf. Proc. IEEE Eng. Med. Biol. Soc..

[34] Lesage S (2002). CARD15/NOD2 mutational analysis and genotype-phenotype correlation in 612 patients with inflammatory bowel disease. Am. J. Hum. Genet..

[35] Hugot JP (2001). Association of NOD2 leucine-rich repeat variants with susceptibility to Crohn’s disease. Nature.

[36] Chamaillard M (2003). Gene-environment interaction modulated by allelic heterogeneity in inflammatory diseases. Proc. Natl. Acad. Sci. USA.

[37] Yu J-W (2007). Pyrin Activates the ASC Pyroptosome in Response to Engagement by Autoinflammatory PSTPIP1 Mutants. Mol. Cell.

[38] Mandey SHL, Schneiders MS, Koster J, Waterham HR (2006). Mutational spectrum and genotype-phenotype correlations in mevalonate kinase deficiency. Hum. Mutat..

[39] Drenth JP (1999). Mutations in the gene encoding mevalonate kinase cause hyper-IgD and periodic fever syndrome. International Hyper-IgD Study Group. Nat. Genet..

[40] Houten SM (2001). Organization of the mevalonate kinase (MVK) gene and identification of novel mutations causing mevalonic aciduria and hyperimmunoglobulinaemia D and periodic fever syndrome. Eur. J. Hum. Genet..

[41] Ozen S, Bilginer Y (2014). A clinical guide to autoinflammatory diseases: familial Mediterranean fever and next-of-kin. Nat. Rev. Rheumatol..

[42] French FMF (1997). Consortium. A candidate gene for familial Mediterranean fever. Nat. Genet..

[43] Wu Z (2015). Association between MEFV Mutations M694V and M680I and Behçet’s Disease: A Meta-Analysis. PLoS One.

[44] Tasliyurt, T., Yigit, S., Rustemoglu, A., Gul, U. & Ates, O. *Common MEFV gene mutations in Turkish patients with Behcet’s disease*. *Gene* **530** (2013). 10.1016/j.gene.2013.08.026 23973724

