# Supplementary material for: Mutational profile of rare variants in inflammasome-related genes in Behçet disease: A Next Generation Sequencing approach
Source: Sci Rep. 2017 Aug 16;7:8453. doi: 10.1038/s41598-017-09164-7 (PMC5559572; doi:10.1038/s41598-017-09164-7)
Supplement: Supplementary file 1 — Supplementary Material [file 41598_2017_9164_MOESM1_ESM.pdf]

Mutational profile of rare variants in inflammasome-related genes in Behçet disease. A Next Generation Sequencing approach

PhD Sergio Burillo-Sanz[1], PhD Marco-Antonio Montes-Cano[1], PhD José-Raúl García-Lozano[1], PhD Lourdes Ortiz-Fernández[1], Norberto Ortego-Centeno[2], Francisco-José García-Hernández[3], Gerard Espinosa[4], Genaro Graña-Gil[5], Juan Sánchez-Bursón[6], María Rosa Juliá[7], Roser Solans[8], Ricardo Blanco[9], Ana-Celia Barnosi-Marín[10], Ricardo Gómez de la Torre[11], Patricia Fanlo[12], Mónica Rodríguez-Carballeira[13], Luis Rodríguez-Rodríguez[14], Teresa Camps[15], Santos Castañeda[16], Juan-Jose Alegre-Sancho[17], PhD Javier Martín[18], PhD María Francisca González-Escribano[1]*.

[1]Department of Immunology, Hospital Universitario Virgen del Rocío (IBiS, CSIC, US), Sevilla 41013, Spain. [2]Department of Internal Medicine, Hospital Clínico San Cecilio, Granada 18003, Spain. [3]Department of Internal Medicine, Hospital Universitario Virgen del Rocío, Sevilla 41003, Spain. [4]Department Autoimmune Diseases, Hospital Universitari Clínic, Barcelona 08036, Spain. [5]Department of Rheumatology, Complejo Hospitalario Universitario A Coruña, A Coruña 15006, Spain

[6]Department of Rheumatology, Hospital Universitario de Valme, Sevilla 41014, Spain

[7]Department of Immunology, Hospital Universitari Son Espases, Palma de Mallorca 07120, Spain. [8]Department of Internal Medicine, Autoimmune Systemic Diseases Unit, Hospital Vall d'Hebron, Universidad Autonoma de Barcelona, Barcelona 08035, Spain.

[9]Department of Rheumatology, Hospital Universitario Marqués de Valdecilla, Santander 39008, Spain. [10]Department of Internal Medicine, Complejo Hospitalario

Torrecárdenas, Almería 04009, Spain. [11]Department of Internal Medicine, Hospital Universitario Central de Asturias, Asturias 33011, Spain. [12]Department of Internal Medicine, Hospital Virgen del Camino, Pamplona 31008, Spain. [13]Deparment of Internal Medicine, Hospital Universitari Mútua Terrassa, Terrassa 08221, Spain. [14]Department of Rheumatology, Hospital Clínico San Carlos, Madrid 28040, Spain. [15]Department of Internal Medicine, Hospital Regional Universitario de Málaga, Málaga 29010, Spain. [16] Department of Rheumatology, Hospital de la Princesa, IIS-Princesa, Madrid 28006, Spain. [17]Department of Rheumatology, Hospital Universitario Doctor Peset, Valencia 46017, Spain. [18]Instituto de Parasitología y Biomedicina "López-Neyra", CSIC, PTS Granada, Granada 18016, Spain.

Corresponding autor: María Francisca González-Escribano, Servicio de Inmunología. HU Virgen del Rocío. 41013 Sevilla. Spain. mariaf.gonzalez.sspa@juntadeandalucia.es. Tel +34955013228 Fax +34955013221.

Table S1. Variants in Inflammasome-related genes identified in Behçet disease patients in the present study.

Mutations	SNP id.	Alleles nbr. N=710	Alleles (MAF) 1KG ALL pops. N=5008	Alleles (MAF) 1KG IBS pop. N=214	Reported pathology	PolyPhen2 functional prediction	SIFT functional prediction
CECR1_Arg49Trp	rs199614299 (**)	2	0 (0)	0 (0)	Unknown	Benign	Damaging
CECR1_Ala247Val	rs750868279 (**)	1	0 (0)	0 (0)	Unknown	Damaging	Tolerated
CECR1_Met309Ile	rs146597836	3	3 (0.0006)	1 (0.005)	Unknown	Benign	Tolerated
CECR1_His335Arg	rs2231495	218	1826 (0.3646)	58 (0.271)	Non-functional Polym	Benign	Tolerated
CECR1_Val349Ile	rs74317375	3	4 (0.0008)	0 (0)	Unknown	Benign	Tolerated
CECR1_Thr360Ala	rs775440641 (**)	2	0 (0)	0 (0)	Sneddon's syndrome	Damaging	Damaging
MEFV_Ala89Thr	rs104895124 (**)	1	0 (0)	0 (0)	FMF	Damaging	Damaging
MEFV_Leu110Pro	rs11466018	1	59 (0.0118)	0 (0)	FMF	Benign	Damaging
MEFV_Gly111Glu	rs751454741 (**)	1	0 (0)	0 (0)	Unknown	Benign	Tolerated
MEFV_Glu148Gln	rs3743930	5	633 (0.1264)	0 (0)	FMF, Functional Polym	Damaging	Damaging
MEFV_Arg202Gln	rs224222	156	681 (0.136)	63 (0.294)	Non-functional Polym	Benign	Tolerated
MEFV_Leu367Val	NA	1	0 (0)	0 (0)	Unknown	Benign	Tolerated
MEFV_Pro369Ser	rs11466023	3	101 (0.0202)	1 (0.005)	FMF	Damaging	Damaging
MEFV_His404Arg	rs755659290 (**)	1	0 (0)	0 (0)	Unknown	Damaging	Damaging
MEFV_Arg408Gln	rs11466024	3	86 (0.0172)	1 (0.005)	FMF	Benign	Tolerated
MEFV_Met582Leu	rs104895165 (**)	1	0 (0)	0 (0)	Recurrent Arthritis, FMF	Benign	Tolerated
MEFV_Ile591Thr	rs11466045	5	22 (0.0044)	2 (0.009)	FMF	Benign	Tolerated
MEFV_Met694Val	rs61752717	2	1 (0.0002)	0 (0)	FMF	Benign	Tolerated
MEFV_Lys695Arg	rs104895094	1	9 (0.0018)	1 (0.005)	FMF	Damaging	Tolerated
MEFV_Val726Ala	rs28940579 (**)	1	0 (0)	0 (0.000)	FMF	Benign	Tolerated
MEFV_Ala744Ser	rs61732874	5	9 (0.0018)	4 (0.019)	FMF	Benign	Tolerated
MEFV_Arg761His	rs104895097 (**)	1	0 (0)	0 (0)	FMF	Benign	Tolerated
MVK_Val5Ala	rs141765653	1	1 (0.0002)	0 (0)	HIDS	Benign	Tolerated
MVK_Ser52Asn	rs7957619	79	380 (0.0759)	33 (0.154)	Non-functional Polym	Benign	Tolerated
MVK_Val80Ile	rs76914224	1	10 (0.002)	0 (0)	HIDS	Benign	Tolerated
MVK_Thr237Ser	rs104895366 (**)	1	0 (0)	0 (0)	HIDS	Damaging	Tolerated
MVK_Val293Met	rs104895356	1	1 (0.0002)	0 (0)	HIDS	Damaging	Tolerated
MVK_Val377Ile	rs28934897 (**)	1	0 (0)	0 (0)	HIDS	Benign	Tolerated
NLRP3_Val198Met	rs121908147	5	20 (0.004)	1 (0.005)	FCAS	Benign	Tolerated
NLRP3_Ile315Val	rs180177501 (**)	1	0 (0)	0 (0)	MAGIC Syndrome	Benign	Tolerated
NLRP3_Arg488Lys	rs145268073	2	1 (0.0002)	0 (0)	FCAS	Benign	Tolerated
NLRP3_Gln703Lys	rs35829419	39	112 (0.0224)	16 (0.075)	FCAS, Functional Polym	Benign	Tolerated
NLRP3_Ser726Gly	rs147946775	1	1 (0.0002)	0 (0)	CINCA/NOMID	Damaging	Tolerated
NOD2_Leu81Val	rs34936594 (**)	1	0 (0)	0 (0)	Unknown	Benign	Damaging
NOD2_Ala140Thr	rs34684955	1	35 (0.007)	0 (0)	CD	Benign	Tolerated
NOD2_Leu248Arg	rs104895423	1	1 (0.0002)	0 (0)	CD	Damaging	Damaging
NOD2_Pro268Ser	rs2066842	61	511 (0.102)	61 (0.285)	Non-functional Polym	Benign	Tolerated
NOD2_Asn289Ser	rs5743271	13	20 (0.004)	2 (0.009)	CD	Benign	Tolerated
NOD2_Arg311Trp	rs104895427	2	4 (0.0008)	0 (0)	CD	Damaging	Damaging
NOD2_Leu349Phe	NA	2	0 (0)	0 (0)	Unknown	Benign	Damaging
NOD2_Arg702Trp	rs2066844	22	72 (0.0144)	12 (0.056)	CD	Damaging	Damaging
NOD2_Arg703Cys	rs5743277	2	5 (0.001)	0 (0)	CD	Damaging	Damaging
NOD2_Val733Leu	NA	1	0 (0)	0 (0)	Unknown	Benign	Tolerated
NOD2_Ala755Val	rs61747625	2	1 (0.0002)	0 (0)	CD	Damaging	Tolerated
NOD2_Arg791Gln	rs104895464	2	1 (0.0002)	0 (0)	Spondylarthropathy	Benign	Tolerated
NOD2_Val793Met	rs104895444	3	1 (0.0002)	0 (0)	CD	Benign	Tolerated
NOD2_Val816Ile	NA	1	0 (0)	0 (0)	Unknown	Benign	Tolerated
NOD2_Met863Val	rs104895447	1	1 (0.0002)	0 (0)	CD	Benign	Tolerated
NOD2_Gly908Arg	rs2066845	7	23 (0.0046)	2 (0.009)	CD	Damaging	Damaging
NOD2_Ala918Asp	rs104895452	1	3 (0.0006)	1 (0.005)	CD	Damaging	Tolerated
NOD2_Val955Ile	rs5743291	62	167 (0.0333)	28 (0.131)	CD, Functional Polym	Benign	Tolerated
PSTPIP1_Thr68Met	rs201872851	2	13 (0.0026)	0 (0)	Unknown	Damaging	Tolerated
PSTPIP1_Val122Ile	NA	1	0 (0)	0 (0)	Unknown	Benign	Tolerated
PSTPIP1_Glu277Asp	NA	1	0 (0)	0 (0)	PAPASH	Benign	Tolerated
PSTPIP1_Asp289His	NA	2	0 (0)	0 (0)	Unknown	Damaging	Tolerated
PSTPIP1_Arg405Cys	rs201253322	2	4 (0.0008)	1 (0.005)	Idiopathic juvenile arthritis	Benign	Tolerated
TNFRSF1A_Pro75Leu	rs4149637	3	141 (0.0282)	0 (0)	TRAPS	Damaging	Tolerated
TNFRSF1A_Arg121Gln	rs4149584	8	30 (0.006)	0 (0)	TRAPS	Benign	Tolerated
TNFRSF1A_His155Tyr	NA	1	0 (0)	0 (0)	Unknown	Benign	Tolerated
TNFRSF1A_Ile199Thr	rs104895247 (**)	2	0 (0)	0 (0)	Unknown	Benign	Tolerated
TNFRSF1A_Arg312Lys	rs200900510	1	6 (0.0012)	0 (0)	Unknown	Benign	Tolerated
TNFRSF1A_Asn336His	NA	4	0 (0)	0 (0)	Unknown	Benign	Damaging

Polym: Polymorphism. NA: not available or SNP undescribed. Unknown: Unavailable information in clinical data bases. 1KG: 1000 genomes, (**): SNP not found in 1000 Genomes Project Phase 3 but found in Exome Aggregation Consortium (ExAC) database.

Table S2. Frequency of 3 *NOD2* loss of function variants in Spanish BD patients and controls

NOD2	p.Asn289Ser		p.Arg702Trp		p.Gly908Arg	
	**BD**	**Controls**	**BD**	**Controls**	**BD**	**Controls**
**Mutated alleles**	13 (0.018)	2 (0.009)	22 (0.03)	12 (0.06)	7 (0.01)	2 (0.01)
**Total tested alleles**	710	214	710	214	710	214
**P-values**	0.36		0.09		0.95	

Controls and their genotypes from 1000 genomes IBS population.

Table S3. Clinical features of BD patients carrying rare variants.

Gene	Nr. Patients	Clinical features					
		**Genital sores (60%)***	**Vascular affection (21%)***	**Digestive affection (16%)***	**Arthritis (42%)***	**SNC affection (18%)***	**Uveitis (54%)***
**CECR1**	11	67%	0%	33%	33%	33%	0%
**MEFV**	32	89%	11%	22%	44%	22%	78%
**MVK**	5	0%	0%	80%	80%	0%	0%
**NLRP3**	9	67%	33%	33%	67%	0%	33%
**NOD2**	40	43%	0%	14%	43%	0%	29%
**PSTPIP1**	8	50%	0%	17%	67%	17%	50%
**TNFRSF1A**	19	67%	33%	33%	33%	33%	67%

For each gene, percentage of patients with rare variants having each clinical manifestation is displayed.
*Percentage of patients with this clinical feature in the BD cohort.
No significant differences were found in any case (Pc>0.05).

A

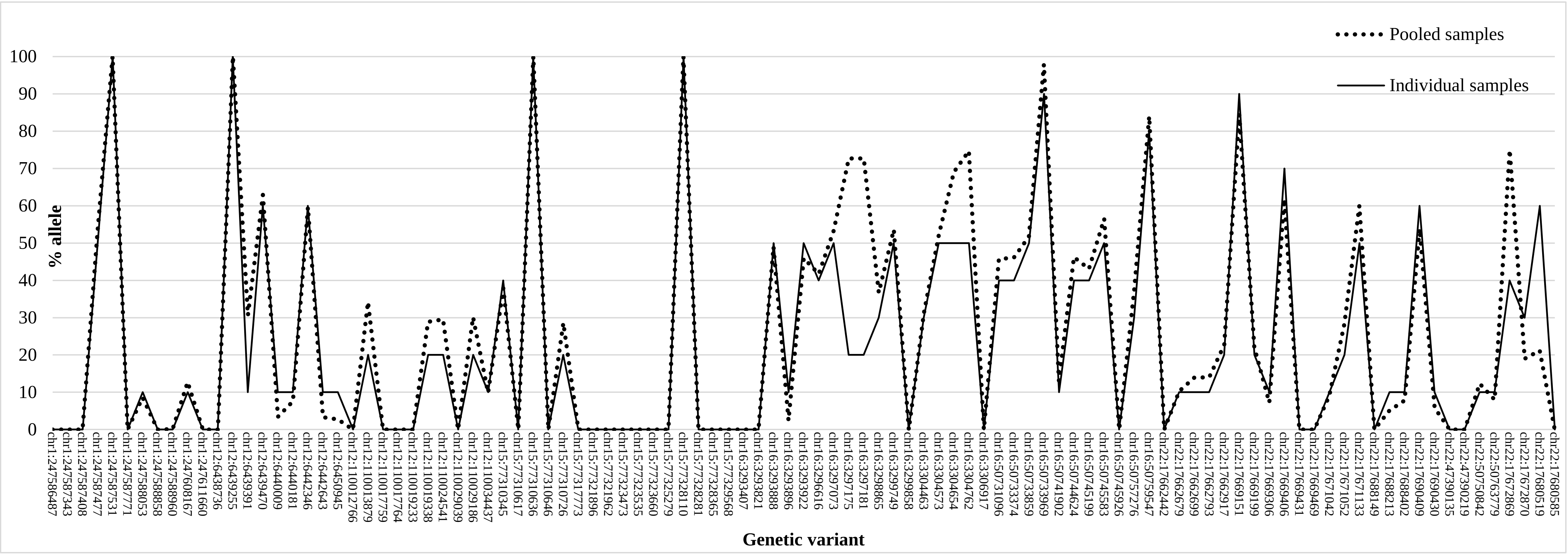

B

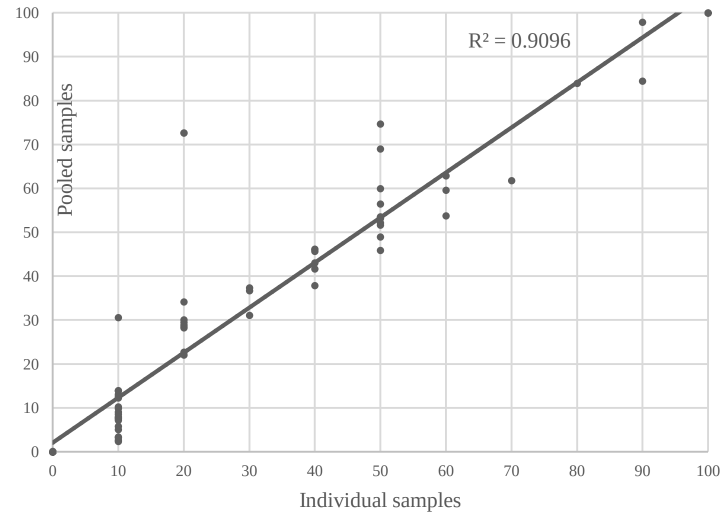

Figure S1. Validation of the method used to quantify alleles in the pooled samples. **A.** Representation of the percentage of each allele in 101 polymorphic positions (listed in the x axis) sequenced in 5 samples, in both ways, individually (continuous line) and pooled (dotted line). **B.** Coefficient of linear correlation (R^2) for the percentage of the alleles quantification in the same quality control.

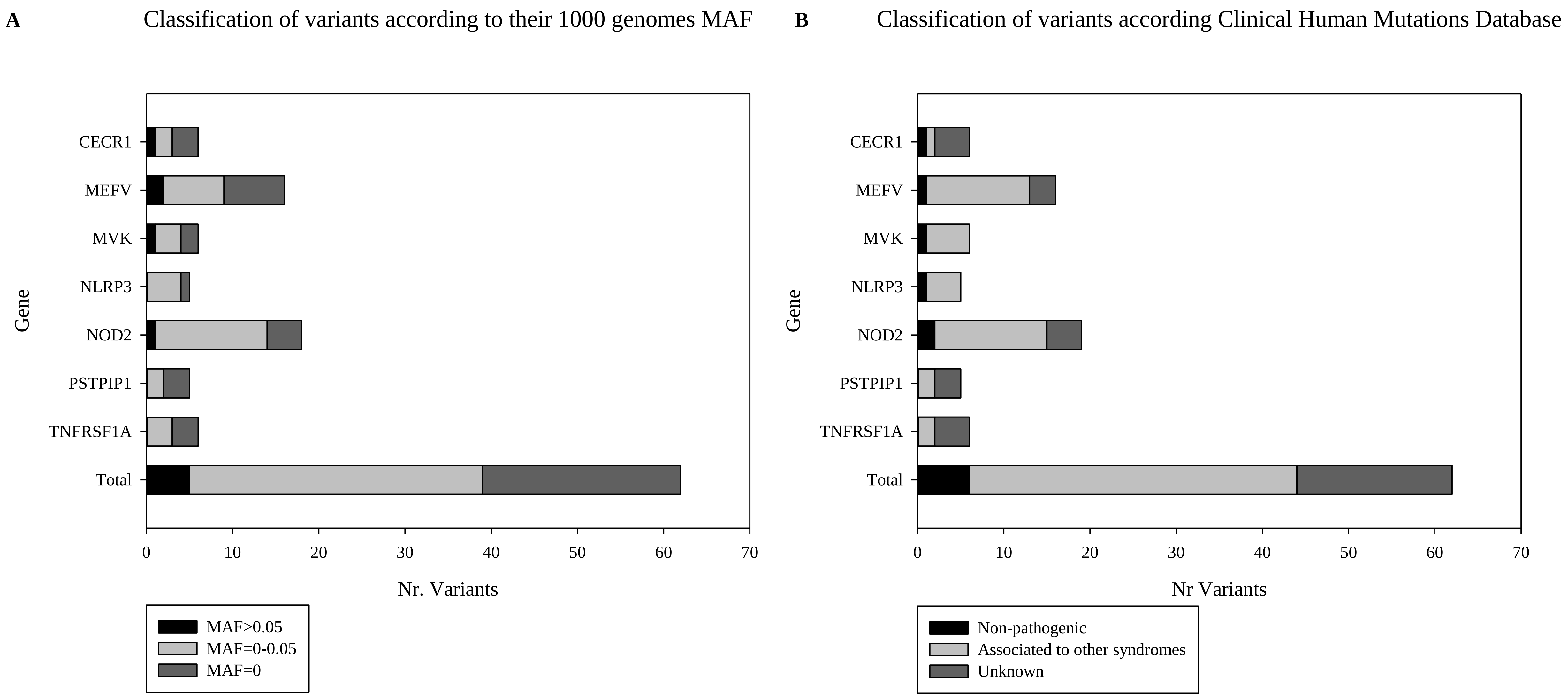

Figure S2. Graphical representation of the number of variants classified in subgroups according to (**A**) their 1000 genomes MAF and (**B**) the information available in clinical human mutation databases.
